# Optimization of contact angle and interfacial tension measurements for fluid/rock systems at ambient conditions

**DOI:** 10.1016/j.mex.2019.07.009

**Published:** 2019-07-12

**Authors:** Wajdi Alnoush, Amr Sayed, Nayef Alyafei

**Affiliations:** Department of Petroleum Engineering, Texas A&M University at Qatar, Qatar

**Keywords:** Interfacial tension (Pendant Drop Method), static contact angle (Sessile Drop Method) and advancing and receding contact angles (Changing Volume Method or Captive-Drop Method), Interfacial tension, Contact angle, Wettability, Optimization, Multi-phase, Reservoir, Immiscible fluids

## Abstract

Quantification of interfacial tension (IFT) and contact angles is essential to characterize reservoir fluid-fluid and rock-fluid interactions. However, these measurements are highly dependent on chemical structure, surface residues, system conditions, flow dynamics and interface interactions. Interfacial interactions are specifically important for secondary and tertiary oil recovery processes where water or brine is injected to recover residual oil through complex multi-phase - rock/brine/oil- interactions Due to the delicate interactions across the interfaces, these systems are subjected to variations and inconsistencies that might be difficult to eliminate, thus, affecting the reliability, reproducibility, and certainty of the data. Therefore, reliable measurements need to eliminate fundamental sources of error and remove surface contaminants. To optimize these measurements, the presented methods take a holistic approach to carefully and reliably optimize the procedure for interfacial tension and contact angle measurements for fluid-fluid and rock-fluid interaction studies at ambient conditions (22.0 ± 2 °C and atmospheric pressure).

• The presented method ensures minimization of impurities that alter interfacial tension measurements;
• Enhanced preparation for tested surfaces for contact angle measurements, and;
• Optimization of procedure at ambient conditions

## Graphical abstract

**Figure:**
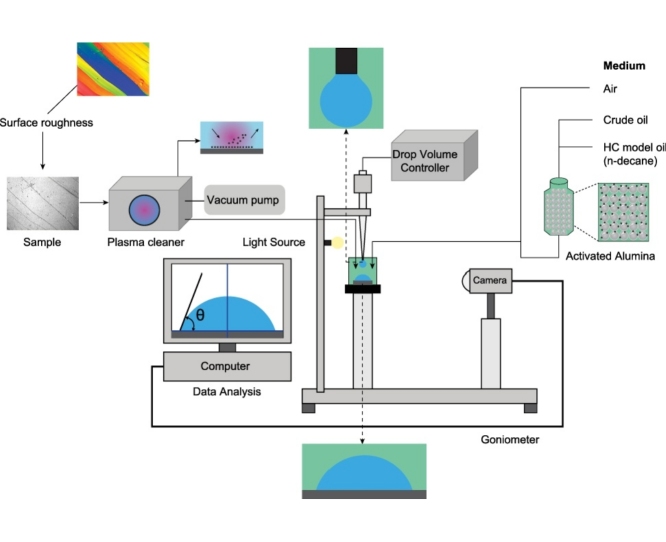

**Specifications Table**

**Table:** 

Subject Area:	Chemistry
More specific subject area:	Improved oil recovery
Method name:	Interfacial tension (Pendant Drop Method), static contact angle (Sessile Drop Method) and advancing and receding contact angles (Changing Volume Method or Captive-drop Method)
Name and reference of original method:	N/A
Resource availability:	N/A

## Method details

### Introduction

Understanding the interactions between immiscible fluids is important in many different fields. For instance, in petroleum reservoirs, which hold large quantities of hydrocarbon resources, the existence of gaseous, aqueous and organic phases creates a multi-phase interaction scheme. Typically, interfaces between immiscible phases are strongly dependent on the nature of phases, chemical structure, saturation profiles, system conditions and flow dynamics. Therefore, it is important to investigate those interface interactions to create a comprehensive understanding of the behavior of reservoir fluids. Such depictions lead to practical and applicable solutions to govern the behavior of reservoir fluids. Reservoirs undergo three main modes of oil recovery through their life cycles. In the first, primary recovery, oil is recovered directly from the reservoir. The second mode is secondary recovery where water or gas are injected to for pressure maintenance and oil displacement. The third and last subsequent mode is tertiary recovery which involves waterflooding or enhanced oil recovery methods through injection of brines and other special fluids [1]. Therefore, interfacial interactions are specifically important for secondary and tertiary oil recovery processes where water or brine is injected to recover residual oil through complex multi-phase - rock/brine/oil- interactions [2].

As essential reservoir rock and fluids parameters, interfacial tension and wettability dictate several aspects associated with oil recovery. The first recognition of the wetting phenomena was by Galileo in the 17^th^ Century while equations to characterize contact angles were first formulated by Young in 1805 [3,4]. Wettability is defined by Craig [5] as the tendency of a fluid to spread on or adhere to a solid surface in the presence of other immiscible fluids. Wettability states are categorized into water-wet, intermediate-wet and oil-wet. Although, theoretical definition of water-wet, oil-wet and intermediate-wet states is based on specific limits of contact angles; <90°, >90° and ≅90° respectively, wettability states of analyzed systems are typically assigned a range of contact angles. Typical ranges are 0-80° for water-wet, 80-100° for intermediate wet and 100–180° for oil-wet [6]. Reservoir rocks can vary in their wettability states ranging from strongly water-wet to strongly oil-wet. The majority of real systems can be characterized as mixed-wet [7]. There has been several direct and indirect methods for the determination of surface wettability of reservoir rocks at micro and molecular scales. These include chromatographic tests, electrokinetic potential measurements, microscopy techniques (such as NMR or AFM) and contact angle measurements [8]. Contact angles used to be measured manually and those measurements were subjected to error and bias from different operators. Thus, the development of automated measurement tools followed for the purpose of improving accuracy and the ability to conduct numerous contact angle measurements through large datasets [9].

Interfacial tension is the energy per unit area of an interface between two phases. The use of pendant drop has been known for the determination of interfacial tension from the shape of the drop. Initially, results from this method were subjected to considerable error due to quality of photographing images and empirical evaluation methods. Lately, developments in electronic data processing and computer based analysis software allowed digitalizing drop images and solving equations of drop contour. Through drop shape analysis, liquid-fluid interfacial tensions are determined. The drop shape analysis method for pendant drop is the most advanced technique for IFT measurements through a large range of pressures and temperatures [10].

Aspects including purification of oil, surface treatment, roughness characterization and procedures of conducting the experiments are addressed in this paper. It is important to highlight that “optimization” does not guarantee accuracy. Optimizing the results for a calcite/brine/n-decane system, for instance, through eliminating sources of error and following proper procedures does not guarantee that the results accurately represent carbonate reservoir systems as other factors and conditions are also in effect. Quantitative accuracy for specific systems is addressed in more detail in other sources in literature.

### System fluids

Interfacial tension and wettability studies predominantly focus on the interface interactions between an oil and an aqueous (brine) phase, as well as a solid phase, as representative systems of interest related to oil recovery. Since gas does not require any preparation procedure, in reservoir studies, and does not commonly form a wetting phase, this method focuses on the two liquid phases (oil/brine). Discussion on the aqueous and oil phases are present in the following sections.

#### Aqueous phase

Waterflooding, as an improved oil recovery (IOR) method, focuses on directing water to displace oil from reservoirs. Therefore, the aqueous phase (brine) plays a major role in the interaction and recovery of the available oil. Waterflooding methods have proven to be inexpensive, advantageous and practical in increasing oil recovery. Complexities arise through the addition of organic compounds, surfactants or polar components to the injected water. The addition of salts, surfactants, foam or polymers aims to alter the brine/oil/rock system by reducing brine/oil interfacial tension and increasing oil mobility and, thus, recovery [11].

#### Oil phase

Conducting studies related to oil recovery and reservoir fluids can use realistic -extracted- crude oil, simplified hydrocarbon model oil (mostly alkanes) or a mixture of model oils. Many studies take n-decane, for instance, as representative model oil to investigate water/oil/rock systems. It is important for these oils to be pure and free from impurities and residues for reliable results suitable for reporting and benchmarking with literature.

**Fig. 1:**
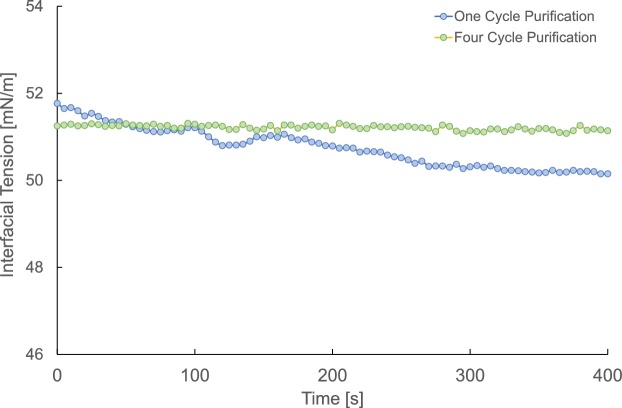
Shows the difference in the interfacial tension readings between a One-Cycle purification and a Four-Cycle purification of n-decane with activated alumina for the n-decane/deionized water system.

In this method, it is recommended to purify model oils using adsorbents to avoid alteration in the water-oil interface caused by impurities. To purify n-decane from oxidized alkanes and other residues, this method uses activated alumina columns. The process entails passing the model oil (n-decane) through the adsorption alumina column for, optimally, at least 4 cycles. The number of cycles might depend on the purity of the model oil and/or the storage environment and duration. Fig. 1 shows the difference in interfacial tension readings between deionized water (produced from Milli-Q® Integral 10 water purification system) and n-decane purified through one and four cycles with activated alumina. The data is obtained from pendant drop interfacial tension measurements using ramé-hart model 500 goniometer. The figure indicates that four cycles of purification result in more consistent and stable readings. This is associated with the complete removal of impurities that might alter the oil/brine or oil/rock interface interactions as opposed to the case with one cycle.

### Interfacial tension

**Fig. 2:**
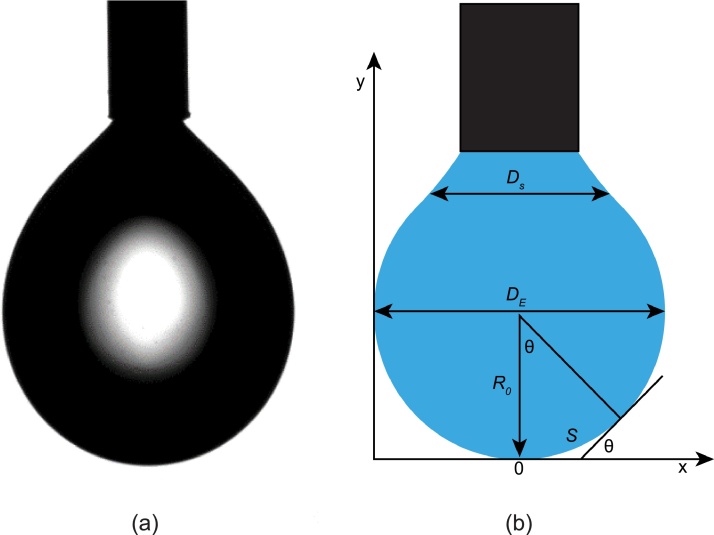
Schematic showing (a) a capture of pendant drop IFT experiment and (b) an illustration the geometrical dimensions of the pendant drop method.

The behavior of the interfacial layer between two immiscible fluids is highly dependent on the species in each phase. There are several ways to characterize and quantify the interfacial tension between two phases. In the laboratory, it is common to use the pendant drop method coupled with computer image analysis. This method is rapid, has low cost and reasonable accuracy [12]. Fig. 2 shows a capture from an IFT experiment along with a schematic of the geometrical dimensions used for the drop shape analysis. In the labeled geometries, S is the distance along the drop profile from the drop apex, $D_{E}$ is the maximum diameter, $D_{s}$ is the diameter at the distance $D_{E}$ (maximum diameter) from the apex and *θ* is the angle between the horizontal and a tangent to the drop profile at a point defined by its *x* and *y* coordinates [12]. Interfacial tension can be evaluated through analysis of the droplet geometry and the difference in densities of the phases as represented by the equation below; full derivation of the equation is described by Hansen and Rødsrud [12]:

(1) $$\sigma =\frac{\Delta \rho g{r\;}^{2}}{\beta }$$

where σ is the interfacial tension [N/m], Δρ is the density difference of the phases [kg/m^3^], g is the gravitational acceleration [m/s^2^], r is the radius of curvature at the drop apex [m], and β is a dimensionless shape parameter. To optimize the measurements of interfacial tension studies it is essential to ensure the following:

• Consistency in test conditions (special considerations to ventilation rates, room temperature).
• Consistency in drop size.
• Solutions are properly mixed to avoid having precipitated crystals that hinder the true interfacial tension.
• Proper device and camera calibration.

*Procedure: Interfacial Tension (Pendant Drop Method)*

1 Change or clean (with deionized water) and dry the micro syringe (used for fluid injection).
2 Pour few milliliters of the aqueous phase into a small beaker.
3 Rinse the micro syringe and the drop volume controller system with the aqueous (brine) phase for 3–5 cycles.
4 Fill the cell with the selected medium (air, purified model oil or crude oil).
5 Insert the micro syringe in the cell until the tip is clearly visible.
6 Switch on the light source.
7 Incrementally inject the liquid until a pendant drop is hanging on the tip of the syringe.
8 Set the intensity of the light such that the contrast between phases is clear.
9 Incrementally increase the volume of the drop until it is at a maximum volume beyond which it would dispatch.

Table 1 below lists obtained interfacial tension measurement for deionized water/n-decane system in comparison to literature values. The results show agreement with literature reported results.

**Table 1:** Interfacial tension measurements for deionized water/n-decane system.

Study	Interfacial Tension [mN/m]
Cai et al. [13]	51.55
Zeppieri et al. [14]	52.30
Georgiadis et al. [15]	51.96
Current study	51.08

### Contact angle

In the context of petroleum reservoirs, the displacement of oil by another phase (through waterflooding for instance) is an essential process for oil recovery. Wettability, as a pivotal parameter, plays a major role in governing this displacement.

As mentioned earlier, a water/oil/rock system can be oil-wet, water-wet, or intermediate-wet, although real fields typically exhibit mixed-wet behavior. Mechanisms governing rock-fluid wettability state include, acid/base interactions, polar interactions, ion binding, and asphaltene precipitation [16]. Regardless of which of these mechanisms dictate the wettability state of the analyzed system, procedures presented in this report ensure optimum measurements for water/oil/rock contact angles.

#### Surface preparation

According to the above discussion, it is essential for rock-fluid wettability studies to be operated at conditions where the rock is well prepared (cleaned from impurities) and characterized. Studies on reservoir rocks (carbonates, sandstones and dolomite) or representative constituent rocks (such as calcite and quartz) are dependent on the wettability state and solid-fluid surface interactions. Thus, it is important to characterize and adjust the surface for optimized results. This is conducted through adjusting the surface roughness and treating it to remove contaminants as discussed in the following subsections.

#### Surface roughness

**Fig. 3:**
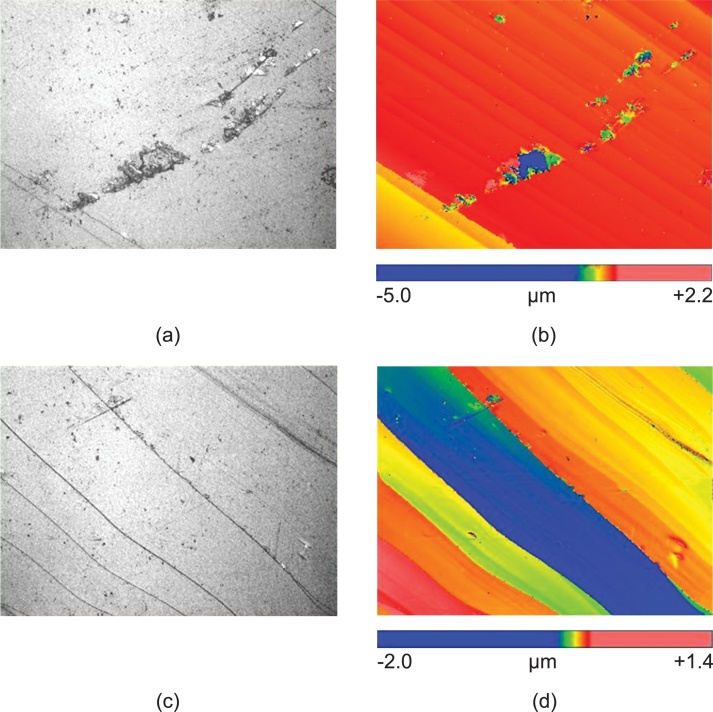
Sample interferometer surface roughness profiles for calcite rocks where (a) and (b) represent a surface with a more homogeneous roughness and (c) and (d) represent a surface with considerable roughness (large peak and color variations).

**Fig. 4:**
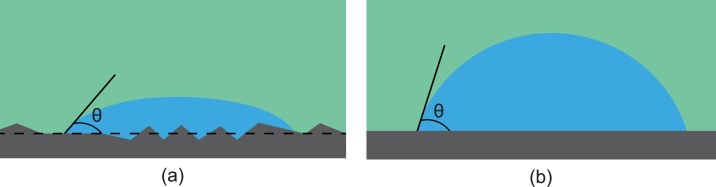
Schematic of a rock/brine/oil system showing the effect of surface roughness on contact angle in a hydrophilic surface when the surface is (a) rough and (b) smooth. (Blue signifies the water phase and green represents the oil phase).

Materials formed by compaction of grains, such as reservoir rocks, display roughness at grain scale [17]. The wetting characteristics of a solid are strongly dependent on the surface roughness [18]. Surface roughness is typically reported as elevations or peak-to-valley differences at the surface and quantified as root mean square of the profile height deviations from the mean line. It is important to measure roughness when using representative mineral rocks (such as calcite and quartz) in reservoir system studies in order to approximate reservoir rock surface conditions. Fig. 3 below represents the effect of roughness on surface profiles for calcite samples. The roughness was quantified using a non-contact white light interferometer (Zygo NewViewTM 7300). The importance of characterizing the surface roughness relies in its effect on the wettability state of the system through altering the contact angle. Hydrophilic surfaces (*θ* < 90°) show increased wettability with increased roughness whereas hydrophobic surfaces (*θ* > 90°) become more hydrophilic (decreased wettability) [17]. Therefore, optimizing contact angle measurements for reservoir studies dictate proper analysis of surface roughness. Fig. 4 illustrates the effect of surface roughness on the contact angle of a hydrophilic surface at a rock/fluid interface.

#### Surface treatment

Plasma is a partially ionized gas consisting of neutral atoms or molecules, ions and electrons. Although the plasma gas would be at near ambient temperature, electrons are at a much higher temperature than neutral gas. The effect of plasma is on the near surface components and does not extend to the bulk of the material. It can clean surfaces leaving no organic residues and is not limited by surface tension such as the case with wet cleaning methods. Surface treatment of samples prior to contact angle measurements eliminates the effect of impurities and surface residues on the contact angle and increases the reliability and reproducibility of the results.

*Procedure: Plasma Cleaning - Harrick Plasma model PDC-002 (230 V)*

*Loading the sample*

1 Dry the sample with a tissue, if wet.
2 Insert the sample into the plasma cleaner chamber with the testing plane facing up.
3 Check that the venting valve is closed.
4 Close and hold the door while switching on the vacuum pump until the door is held in place.

*Generating the plasma*

1 Switch on the Radio Frequency (RF) power.
2 Select the desired RF power level (medium or high is recommend; maximum RF power is 30 W).
3 Observe the plasma glow (pink-purple for air) through the plasma cleaner window.
4 Adjust the process gas inlet valve to maximize the plasma intensity.
5 Keep the sample exposed to intense plasma for a minimum of 1.5 min. (time required to guarantee removal of contaminants based on past experiments conducted on the same rock sample)

*Venting the vacuum chamber*

6 Turn off the RF power.
7 Close the air intake valve.
8 Open the venting valve until the chamber is at atmospheric pressure.

**Fig. 5:**
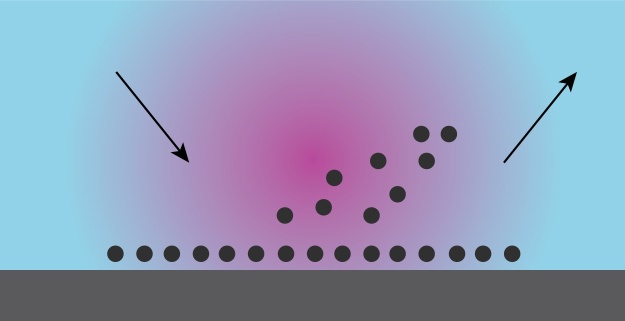
Schematic illustrating surface treatment by plasma gas. The plasma cleans all chemical residues and surface contaminants.

Fig. 5 illustrates the surface treatment process using plasma gas.

#### Static and advancing/receding contact angle

*Procedure: Static Contact Angle (Sessile Drop Method) - ramé-hart model 500*

*Pre-check*

1 Enclose the goniometer with a box or curtains to avoid the effect of external light, ventilation and external contaminations.
2 Check that the device is on a stable laboratory bench with no associated vibrations.

*Static Contact Angle Test*

1 Properly clean the quartz cell (container) with water followed by acetone then dry with pressurized air.
2 Place the surface treated sample at the bottom of the cell with a wide spatula or tongs. (avoid any interaction of skin or other objects with the plane to be tested)
3 Fill the cell with the selected medium (air, purified model oil or crude oil)
4 Switch on the light source.
5 Insert the micro syringe in the cell until the tip is slightly *above* the sample surface.
6 Incrementally dispose the liquid (brine) until a drop is released and dispatched from the tip of the syringe.
7 Set the intensity of the light such that the contrast between phases is clear.
8 Adjust the focus knob until the drop-medium interface is clearly defined.
9 Adjust the tilt of the cell holder using the built-in leveler (before adding the sessile drop) until the tilt is less than 0.3%.
10 Locate the interface lines on the computer software (DROPimage Advanced) to indicate the droplet-solid interface and the middle of the droplet.
11 Take equidistant readings for 5–10 min and report the average value for the static contact angle (approximately 5–20 readings).

**Fig. 6:**
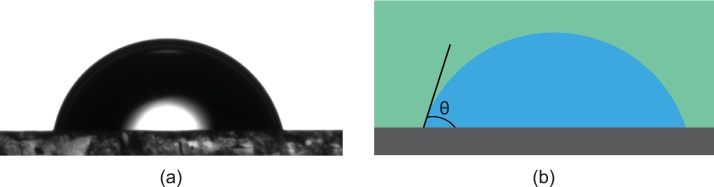
Schematic of a calcite/brine/n-decane system showing (a) a capture of an experimental sessile drop contact angle measurement as captured from a ramé-hart (model 500) goniometer and (b) an illustration of the sessile drop method.

Fig. 6 illustrates the static contact angle measurement using the sessile drop method.

*Procedure: Advancing and Receding Contact Angles (Volume Changing Method) - ramé-hart model 500*

*Pre-check*

1 Enclose the goniometer with a box or curtains to avoid the effect of external light, ventilation and external contaminations.
2 Check that the device is on a stable laboratory bench with no associated vibrations.

*Advancing and Receding Contact Angle Test*

1 Follow steps 1–5 from the static contact angle procedure.
2 Insert the micro syringe in the cell until the tip is *near* the sample surface.
3 Set the intensity of the light such that the contrast between phases is clear.
4 Incrementally dispose the liquid (brine) until a drop is hanged from the tip and adhered to the surface. The drop should remain attached to the micro syringe to subsequently adjust the drop volume.
5 Adjust the focus knob until the drop-medium interface is clearly defined.
6 Set the volume increment from the drop volume control to the desired value (˜0.25 μL).
7 Adjust the tilt of the cell holder using the built-in leveler (before adding the sessile drop) until the tilt is less than 0.3%.
8 Start incremental release of the fluid from the syringe and record contact angle measurements until the contact angle reaches a maximum (advancing contact angle).
9 Reverse the process by incrementally withdrawing fluid from the drop and recording contact angle measurements until the contact angle no longer decreases (Receding contact angle).

**Fig. 7:**
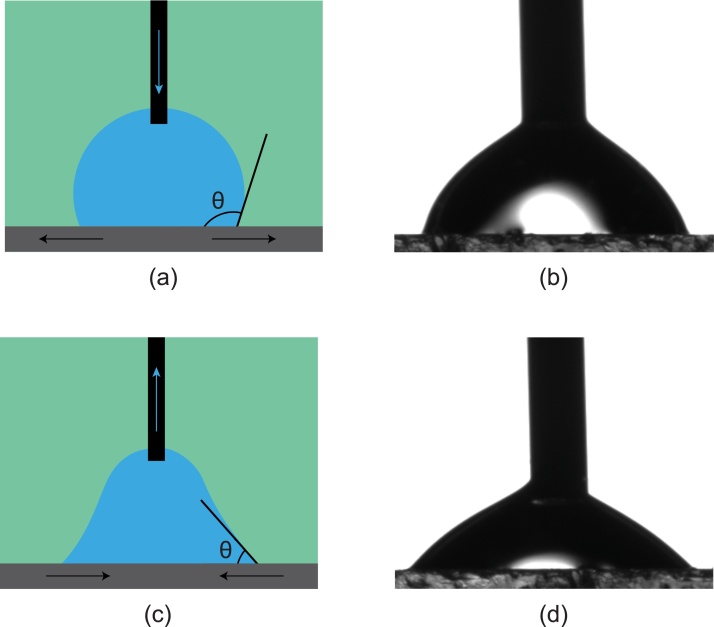
Schematic of a calcite/brine/n-decane system showing (a) and (c) as illustrations for the advancing and receding contact angle using the volume changing method, and (b) and (d) as captured from a ramé-hart (model 500) goniometer experiment on advancing and receding contact angles.

Fig. 7 illustrates the advancing and receding contact angle measurements using the volume changing method.

### Conclusion

It is important to optimize measurements for contact angle and interfacial tension for reliable, reportable and benchmarking worthy studies in reservoir fluids. Due to the dependence of these tests on interactions at the interface between phases, these interactions are to be properly characterized and quantified through optimized testing setups. Elimination of sources of error and impurities is vital to produce reliable and reproducible results. The presented method tackles primary aspects associated with interfacial tension and contact angle measurements and procedures that promise optimized results.
